# Beyond stomatal development: SMF transcription factors as versatile toolkits for land plant evolution

**DOI:** 10.1017/qpb.2024.6

**Published:** 2024-05-31

**Authors:** Yuki Doll, Hiroyuki Koga, Hirokazu Tsukaya

**Affiliations:** 1 Division of Biological Sciences, Graduate School of Science and Technology, Nara Institute of Science and Technology, Nara, Japan; 2 Department of Biological Sciences, Graduate School of Science, The University of Tokyo, Tokyo, Japan

**Keywords:** co-option, stomata, seta, air pore, Marchantia polymorpha

## Abstract

As master transcription factors of stomatal development, SPEECHLESS, MUTE, and FAMA, collectively termed SMFs, are primary targets of molecular genetic analyses in the model plant *Arabidopsis thaliana*. Studies in other model systems identified SMF orthologs as key players in evolutionary developmental biology studies on stomata. However, recent studies on the astomatous liverwort *Marchantia polymorpha* revealed that the functions of these genes are not limited to the stomatal development, but extend to other types of tissues, namely sporophytic setal and gametophytic epidermal tissues. These studies provide insightful examples of gene-regulatory network co-opting, and highlight SMFs and related transcription factors as general toolkits for novel trait evolution in land plant lineages. Here, we critically review recent literature on the SMF-like gene in *M. polymorpha* and discuss their implications for plant evolutionary biology.

## Graphical abstract

**Figure:**
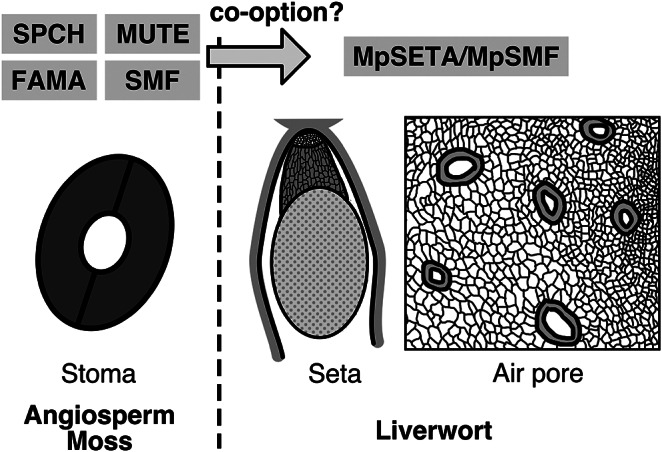

Stomata, gas-exchanging structures resembling a mouth on the surface of shoots, influence various aspects of plant survival and growth. They are good targets for quantitative studies because many sophisticated methods are available to investigate their morphology and functions quantitatively (Kuan et al., 2022). Recent research has also focused on how stomatal traits have evolved during land plant evolution. Fossil and phylogenetic evidence suggest that stomata originated once in early land plants (Edwards et al., 1998; Harris et al., 2020) and then diversified in each lineage (Clark et al., 2022). The highly efficient stomata of grasses represent a good example of such diversification (Franks & Farquhar, 2007; Nunes et al., 2022; Raissig et al., 2017). Many evolutionary developmental biology (evo-devo) studies on stomata have focused on a group of stomatal key transcription factors (TFs) termed SMF (SPEECHLESS/MUTE/FAMA). Recent studies (Chang et al., 2023; Moriya et al., 2023) highlighted the potential for SMFs as versatile toolkit genes shared by all major lineages of land plants; the land plants seem to have utilized SMFs for evolving various cellular traits, including stomatal cells and other cell types. Interestingly, the main material used in these studies was a stomataless liverwort. This *Insights* article focuses on these studies broadening our understanding of SMF functions and discusses the roles that SMFs have played during land plant evolution.

SMFs are TFs belonging to the Ia subfamily of the basic helix–loop–helix (bHLH) family. The acronym ‘SMF’ is derived from the three key TFs mediating stomatal development in the model plant *Arabidopsis thaliana* (Arabidopsis), SPEECHLESS (SPCH), MUTE, and FAMA. These three TFs regulate distinct steps in stomatal development (Figure 1A), together with their common heterodimerization partners INDUCER OF CBF EXPRESSION1/SCREAM (ICE1/SCRM) and SCRM2, and the loss-of-function *spch*, *mute*, and *fama* mutants completely lack functional stomata (Kanaoka et al., 2008; MacAlister et al., 2007; Ohashi-Ito & Bergmann, 2006; Pillitteri et al., 2007). SMFs are widely conserved in the genomes of land plants (Harris et al., 2020; MacAlister & Bergmann, 2011; Ran et al., 2013), and their functions in stomatal development are well conserved in species studied to date (Chater et al., 2016; Liu et al., 2009; Ortega et al., 2019; Raissig et al., 2016, 2017), which highlights their importance in the evolution of stomatal development.

Studies on bryophytes have provided important insights into the evolution of stomatal development. Bryophytes are characterized by their lifecycle dominated by the haploid gametophytic phase, during which no stomata are produced. Bryophyte stomata are only present on the surfaces of diploid sporophytic organs and appear to function in carbon uptake and/or sporophyte drying to facilitate spore dispersal in hornworts and mosses (Chater et al., 2016; Kubásek et al., 2021). A study in the model moss *Physcomitrium patens* revealed that PpSMF1 and PpSCRM1, orthologs of angiosperm SMFs and SCRMs, respectively, form a heterodimer to regulate stomatal development in this species (Chater et al., 2016). This deep functional conservation supports the idea that the origin of the gene regulatory network for stomatal development dates back at least to the common ancestor of bryophytes and tracheophytes.

**Figure 1.**
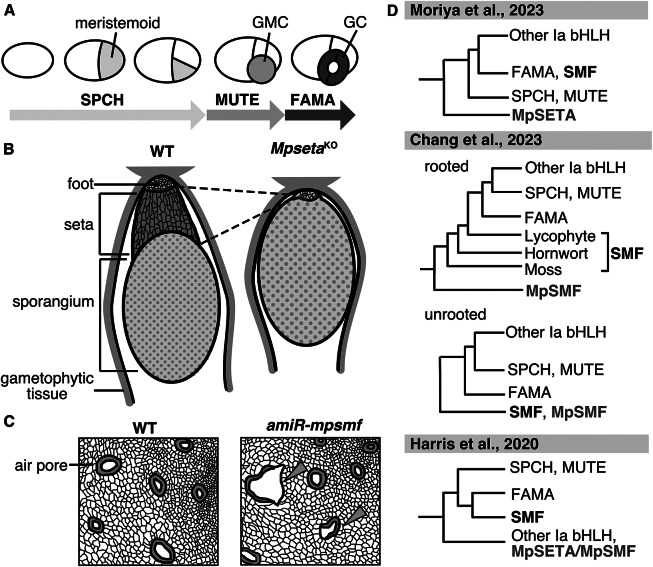
Functions and phylogeny of the SMF-like gene in *M. polymorpha*. (A) Schematic view of the function of SMFs in Arabidopsis stomatal development. SPCH regulates establishment and divisions of stomatal stem cells termed meristemoids. MUTE terminates the meristemoid division and induces its differentiation into the guard mother cell (GMC). FAMA regulates the symmetric division of the GMC to produce a pair of guard cells (GCs). (B) A defective phenotype of setal development in the *Mpseta* ^KO^ line. In the knockout line, the sporophyte lacks the seta, a stalk-like tissue comprising elongated cells (Moriya et al., 2023). (C) A defective phenotype in the gametophytic epidermis of the *amiR-mpsmf* line. Some malformed air pores (arrowheads) are observed in the knockdown line (Chang et al., 2023). (D) Schematic phylogenetic trees of subfamily Ia bHLHs proposed by different groups (Chang et al., 2023; Harris et al., 2020; Moriya et al., 2023). Note that the positions of MpSETA/MpSMF (shown in purple) and SMFs from mosses, hornworts, and lycophytes (shown in bold type) are inconsistent among these trees.

While hornworts and mosses have provided considerable insight into the evolution of stomata (Merced & Renzaglia, 2017), the third lineage of bryophytes, liverworts, appears to remain silent on the matter. Liverworts seem to have lost the ability to form stomata and produce none on gametophytic and sporophytic organs. Some liverworts, including the model species *Marchantia polymorpha*, have instead evolved a gas-exchanging opening on the thallus known as an ‘air pore’ (Harris et al., 2020; Villarreal et al., 2016). In contrast to stomata, which are simple single-cell layered valves composed of two guard cells, air pores themselves are complex multi-cell layered structures with four tiers of multiple cells surrounding a central hole. Just as stomata connect the outside atmosphere to the intercellular air space known as the substomatal cavity, air pores are connected to the intercellular cavity, termed the air chamber, and appear to control gas exchange and water vapor loss (Shimamura, 2016). It is interesting to consider what happened to the gene regulatory network for stomatal development when liverworts lost stomata and acquired air pores.

Moriya et al. (2023) explored this question, focusing on liverwort genes that were sister to all other subfamily Ia bHLHs in their phylogenetic tree. The *M. polymorpha* protein in this group can partially rescue the *mute* mutant phenotype when expressed in Arabidopsis and possesses conserved functional domains previously characterized in angiosperm SMFs. Knocking out this *M. polymorpha* gene, which the authors named *MpSETA*, revealed that the resulting mutant exhibited a defect in the development of sporophytic setal tissue (Figure 1B). The seta is a stalk-like tissue found in mosses and liverworts that comprises files of elongated cells and connects the sporangium and the foot of the sporophyte. In the later stages of sporophyte development, elongation of setal cells thrusts the mature sporangium outside the surrounding gametophytic tissue, facilitating spore dispersal (Shimamura, 2016). *MpSETA* promoter activity was detected in the developing setal region, and the *Mpseta^KO^* mutant completely lacked elongated setal cells (Figure 1B). Thus, it was concluded that MpSETA is a pivotal regulator of setal development. The authors also showed that mutants of *MpICE2*, an *M. polymorpha* ortholog of *ICE1/SCRM*, exhibited defective setal development. MpSETA and ICE1/SCRM were shown to interact with each other, indicating that heterodimerization of SMF and ICE1/SCRM is conserved in liverworts. However, the function of the heterodimer seems to have shifted, or been co-opted, from stomatal cell differentiation to setal cell differentiation. Because the seta is only found in mosses and liverworts, the authors argued that the co-opting might date back to the common ancestor of these two lineages. As pointed out by the authors, further molecular studies on the setal development of mosses are needed to draw a conclusion. While studies in *P. patens* have investigated sporophytic development and the roles of the SMF ortholog in this moss, none of these studies refer to setal phenotypes of the mutants (Caine et al., 2020; Chater et al., 2016). In any case, the setal function of MpSETA and MpICE2 is another interesting example of evolutionary biology in which evolutionary novelty has been achieved by co-opting preexisting genetic modules (DiFrisco et al., 2023).

Chang et al. (2023) provided another view on these genes. They constructed a phylogenetic tree slightly different from that of Moriya et al. (2023) and regarded *MpSETA* as a genuine ortholog of SMFs, thus naming the gene *MpSMF*. In line with the preceding report, Chang et al. showed that the product of this gene can interact with the ICE1/SCRM ortholog and partially rescue the *mute* phenotype in Arabidopsis. However, unlike Moriya et al., they reported the gametophytic phenotypes of the mutants. Interestingly, the *amiR-mpsmf* line with reduced *MpSMF* expression and the knockout line of the ICE1/SCRM orthologs both exhibited defective developmental phenotypes in the gametophytic epidermis, including the reduced density and malformation of air pores (Figure 1C). They also noted that the global over-expression of *MpSMF* resulted in a complete loss of air chambers, though with other defects in multiple gametophytic tissues. Based on these results, the authors argued that these genes are involved in gametophytic development in *M. polymorpha*. Before this report was published, the known functions of SMF TFs had been limited to the sporophytic generation (Chater et al., 2016; Moriya et al., 2023). If the functions of SMFs in *M. polymorpha* have evolved to extend to gametophytic generation, especially in the air pore that acts as a liverwort substitute for stomata, the gene family would serve as an insightful evolutionary model for tracing transgenerational co-option of gene regulatory networks.

However, the two papers convey contradictory arguments that should be tested in future studies. Firstly, they present conflicting views on the expression and function of *MpSETA/MpSMF* in gametophytic tissues. Whereas Chang et al. detected the activity of *MpSETA*/*MpSMF* promoter in the thallus, Moriya et al. detected promoter activity only in one gametophytic tissue (young antheridia) and concluded that its expression was absent or extremely low in most gametophytic tissues based on public RNA sequencing datasets. Chang et al. (2023) did not measure the absolute expression level of the gene normalized against an internal standard; they only measured the relative expression level relative to WT in their quantitative PCR analyses (Fig. S2C in Chang et al., 2023). Therefore, it remains unclear whether the expression of this gene in gametophytic tissues is relevant to morphogenesis as in sporophytic tissue. Since the reported phenotypes in the gametophyte epidermis are not as obvious as those in the setal tissue (Figure 1B and C), interpretation requires a more careful look at the expression dynamics in the gametophyte generation.

The other major discrepancy is that the phylogenetic position of MpSETA/MpSMF is inconsistent in the two papers (Figure 1D). The tree in Moriya et al. places MpSETA/MpSMF in the position sister to all other subfamily Ia bHLHs, including both SMF and less well-characterized members such as MYC-TYPE TRANSCRIPTION FACTOR 70 (MYC70; AtbHLH70) (Ohta et al., 2018) and TARGETS UNDER ETTIN CONTROL 1 (TEC1; AtbHLH94) (Simonini et al., 2017). In their tree, SMF from the other bryophytes and lycophytes are grouped with angiosperm FAMA, as previous studies suggested (Harris et al., 2020; Ran et al., 2013), but not with MpSETA. On the other hand, Chang et al. constructed a tree in which MpSETA/MpSMF, moss SMF, hornwort SMF, lycophyte SMF, FAMA, and the clade comprising angiosperm SPCH and MUTE are all paraphyletic (Figure 1D). They also showed an unrooted tree in which MpSETA/MpSMF and its liverwort orthologs form a monophyletic group with moss SMF. Another previous study (Harris et al., 2020) constructed a tree with a much larger number of sequences and grouped MpSETA/MpSMF with uncharacterized bHLHs from liverworts and gymnosperms (Figure 1D). However, Chang et al. pointed out that the study of Harris et al. included some ‘bryophyte’ sequences resulting from contamination or miss-annotation. As bootstrap values in phylogenetic trees constructed by Moriya et al. and Chang et al. are both not very high, the precise phylogenetic position of MpSETA/MpSMF should be confirmed by more careful examination and quantitative assessment of the reliability of trees with appropriate rooting.

Is MpSETA/MpSMF a direct ortholog of SMFs of other bryophytes? Importantly, the two groups both noted that MpSETA/MpSMF could rescue neither *spch* nor *fama* mutants of Arabidopsis (Chang et al., 2023; Moriya et al., 2023). These results obscure the hypothetical one-to-one orthology, as *P. patens* SMF can partially rescue not only Arabidopsis *mute* but also *fama* mutants (MacAlister & Bergmann, 2011). The specificity of the partial rescue of *mute* by MpSETA/MpSMF should be carefully examined by checking whether other relative bHLHs from *M. polymorpha* might also rescue the *mute* phenotype because MUTE is the shortest protein with the simplest structure among the three SMFs in Arabidopsis (Davies & Bergmann, 2014). Then, should we regard MpSETA/MpSMF as the earliest diverging member of Ia bHLH not directly related to bryophyte SMFs, as in the tree of Moriya et al.? One potential concern arises from the fact that the amino acid sequences of the MpSETA/MpSMF clade proteins are highly divergent from other Ia bHLHs (Chang et al., 2023; Moriya et al., 2023). Rapid evolution in the MpSETA/MpSMF clade could cause long-branch attraction to the outgroup (Kinene et al., 2016) and might wrongly place these genes as sisters of other Ia bHLHs. Denser sampling in liverworts and other bryophytes would help select suitable taxonomic units for tree construction in future studies. Only after constructing a reliable tree, can we infer the ancestral function of subfamily Ia bHLHs from phylogeny. Here, the function of currently less well-characterized members of the family should also be considered because some were shown to interact with ICE1/SCRM (Ohta et al., 2018). In addition, attention should be paid to the possibility that some ancestral genes were lost during evolution because stomata-related genes are prone to be lost in astomatous taxa in both tracheophytes and bryophytes (Hu et al., 2023; Olsen et al., 2016). The precise identity of MpSETA/MpSMF could be determined after further experimental and phylogenetic validation.

Despite these apparent discrepancies, the two studies in *M. polymorpha* strongly support the idea that SMFs and related bHLHs act as versatile toolkit genes for land plant evolution. In seed plants, FAMA regulates the formation of myrosin cells, Brassicales-specific idioblasts (Li & Sack, 2014; Shirakawa et al., 2014), which provides another example of SMF co-option. We recently identified the interspecific differences in the spatiotemporal patterns of SPCH and MUTE expression underlying the diversification of stomatal development patterns in the genus *Callitriche* (Plantaginacea) (Doll et al., 2021a, 2021b, 2023). This shows that SMFs also play a pivotal role in evolution at the species level. Overall, accumulating knowledge on this group of TFs provides significant insight into trait evolution in plants. Another revelation from the studies on MpSETA/MpSMF is the potency of a model system without a specific trait for elucidating the evolutionary history of that trait and the gene regulatory networks behind it. As beautifully presented in a study on vascular development regulators in the non-tracheophyte *P. patens* (Xu et al., 2014) and a study on fin-to-limb transition in limbless zebrafish (Hawkins et al., 2021), the absence of a structure offers an opportunity to trace the genetic changes accompanying the acquirement or loss of that structure. The studies in stomataless *M. polymorpha* remind us of the importance of studying the absence of traits for reconstructing the evolutionary history.

## Data Availability

No data or code were developed for this manuscript.
