# Supplementary material for: Beyond stomatal development: SMF transcription factors as versatile toolkits for land plant evolution
Source: Quant Plant Biol. 2024 May 31;5:e6. doi: 10.1017/qpb.2024.6 (PMC11363000; doi:10.1017/qpb.2024.6)
Supplement: Peer review history

# Editor report: Recommendation: Beyond stomatal development: SMF transcription factors as versatile toolkits for land plant evolution — R0/PR2

**DOI:** 10.1017/qpb.2024.6.pr2

**Affiliations:** Life Science Systems, Technische Universität München, Germany

Dear authors,

I am pleased to inform you that the manuscript was reviewed by four reviewers (a team of two (junior/senior), and two other independent reviewers) demonstrating the interest in your work. All reviewers and myself found the manuscript of interest and a worth contribution to QPB. Please find a list of minor comments to be addressed in a revised version.

Sincerely

Aurelien Tellier

# Editor report: Decision: Beyond stomatal development: SMF transcription factors as versatile toolkits for land plant evolution — R0/PR3

**DOI:** 10.1017/qpb.2024.6.pr3

**Affiliations:** RDP, ENS de Lyon, France

No accompanying comment.

# Editor report: Recommendation: Beyond stomatal development: SMF transcription factors as versatile toolkits for land plant evolution — R1/PR5

**DOI:** 10.1017/qpb.2024.6.pr5

**Affiliations:** Life Science Systems, Technische Universität München, Germany

Dear authors,

I am pleased to accept your revised manuscript. The reviewers have some last final comments to do before you submit the final version of the manuscript.

Best,

Aurelien Tellier

# Editor report: Decision: Beyond stomatal development: SMF transcription factors as versatile toolkits for land plant evolution — R1/PR6

**DOI:** 10.1017/qpb.2024.6.pr6

**Affiliations:** RDP, ENS de Lyon, France

No accompanying comment.
